# Optimizing antibiotic prophylaxis in the surgical setting of an ambulatory hospital for patients with penicillin allergy labels: a quality improvement initiative

**DOI:** 10.1017/ash.2026.10762

**Published:** 2026-06-23

**Authors:** Cassandra Elias, Hazra Chowdhury, Rebecca Wright, Ashley Graham, Karen Devon, Arun Prasad, Lisa Lin, Cameron Thomas, Mira Maximos

**Affiliations:** 1 Women’s College Hospital, Canada; 2 UHN: University Health Network, Canada

## Abstract

At a Canadian ambulatory hospital, a quasi-experimental quality improvement initiative evaluated penicillin-allergy education modules using pre–post surveys. Among 61 clinicians, Module 1 was not associated with change in penicillin allergy label-clinical impact knowledge. Modules 2 and 3 improved allergy-history, severity-assessment, and prophylaxis-prescribing preparedness (*P* < .01). Acceptability was high: documentation tool 92%, algorithm 88%.

## Introduction

Penicillin allergy labels (PALs) are amongst the most commonly reported drug allergies, affecting approximately 10% of individuals worldwide; however, fewer than 1% are truly allergic.^1^ In surgical settings, inaccurate PALs may impact antibiotic prophylaxis decisions and increase use of second-line agents instead of cefazolin, the preferred first-line option for surgical site infection (SSI) prevention.^2,3^

This study followed a 2021–2022 audit of vancomycin and clindamycin use for surgical antibiotic prophylaxis (SAP) at Women’s College Hospital (WCH) which found that 20% of patients receiving second-line antibiotic prophylaxis may have been eligible for cefazolin. In response, our team synthesized evidence on PAL risk-stratification strategies and selected an algorithm for application in the WCH surgical department.^4^ This included tools such as the ACCEPT algorithm, which improved first-line antibiotic prescribing following implementation.^4^ However, prior studies did not assess end-user experience or satisfaction, and, were not conducted in ambulatory settings.^4^

After these findings were presented at WCH, an interdisciplinary Antimicrobial Stewardship (ASP) Working Group was formed. The group implemented a quality improvement (QI) initiative to develop a perioperative workflow addressing gaps in clinician knowledge, acceptability, and preparedness to risk-stratify PALs amongst patients requiring SAP at WCH.

## Methods

This single-center QI study (APQIP Board Approval #2024-0028-P) was implemented at WCH, an ambulatory hospital in Canada, between November 2024–February 2025.

The working group implemented 3 initiatives to improve allergy documentation, PAL assessment, and antibiotic prophylaxis selection. First, we conducted a narrative review of risk-stratification interventions for SAP in patients with PALs to select an implementable algorithm. The Allergy Clarification for Cefazolin Evidence-based Prescribing Tool (ACCEPT) developed by Sunnybrook Health Sciences Centre, was selected and modified.^4^ Second, a standardized allergy documentation tool was developed. Third, three educational modules with pre and post surveys assessed clinician knowledge, preparedness, and tool acceptability (Table 1). Modules 1 and 2 were available to all surgical staff and addressed clinical impact of PALs and allergy documentation. Module 3, tailored to prescribers and pharmacists, guided antibiotic selection using the modified ACCEPT.

**Table 1.** Module survey information

Module	Question	Timing	Response format	Target audience	Statistical test
Module 1: Impact of penicillin allergy labels	Most reported penicillin allergies are true hypersensitivity reactions.	Pre and Post	True/False	All surgical services staff	McNemar’s test
	Once a patient develops a penicillin allergy, they will always have this allergy.				
	The presence of a penicillin allergy label is associated with an increased risk of surgical site infections.				
Module 2: Allergy documentation	Did you receive prior education on gathering a comprehensive drug allergy history?	Pre only	Yes/No	All surgical services staff	Descriptive statistics
	Do you feel prepared to gather a comprehensive drug allergy history?	Pre and Post	10-point Likert scale (very unprepared—very prepared)		Paired *t*-test or Wilcoxon signed rank test^b^
	Do you feel the SmartPhrase allergy history tool is user-friendly?	Post only	Yes/No		Descriptive statistics
Module 3: Risk stratification algorithm	Did you receive prior education on prescribing antibiotics in patients with drug allergies?	Pre only	Yes/No	Clinicians^a^	Descriptive statistics
	Do you feel prepared to assess the severity of a reported penicillin allergy?	Pre and Post	10-point Likert scale (very unprepared—very prepared)		Paired *t*-test or Wilcoxon signed rank test^b^
	Do you feel prepared to prescribe surgical antibiotic prophylaxis for a patient with a penicillin allergy?	Pre and Post			
	Cefazolin can be considered in a patient with a history of anaphylaxis to penicillin.		True/False		McNemar’s test
	Do you feel the risk stratification algorithm tool is user-friendly?	Post only	Yes/No		Descriptive statistics

a Clinicians include surgeons, anesthesiologists, pharmacists, learners, and trainees.

b Statistical test selection based on normality assessment via histogram visualization.

Modules and surveys were administered through REDCap. Surveys focused on knowledge, acceptability, and preparedness using a 10-point Likert scale and true/false question formats. The primary outcome was change in knowledge and preparedness amongst paired responses. Secondary outcomes included tool acceptability and qualitative reflections. Statistical analyses were performed using RStudio. Descriptive analyses summarized sample sizes and professional roles. For paired binary data, McNemar’s test assessed changes in proportions of correct or positive responses between pre and postmodule surveys. For paired preparedness scores, medians, interquartile ranges and Wilcoxon signed rank (WSR) test were used due to non-parametric data. Qualitative feedback was analyzed using inductive thematic analysis.

## Results

Sixty-one participants (nurses 37.7%; surgeons 18%; pharmacists 16.4%; pharmacy technicians 9.8%; others 9.8%; anesthesiologists 8.2%) initiated the modules with various levels of completion.

### Clinical impact of PAL

In module 1 (n = 31 paired responses), none of the response changes were statistically significant (see table 2). Questions include: “Most reported penicillin allergies are true hypersensitivity reactions” (87% to 84% correct pre and post intervention, *P* = 1.00); “Once a patient develops a penicillin allergy, they will always have this allergy” (87% to 94% correct pre and post intervention, *P* = .48); “ The presence of a penicillin allergy label is associated with an increased risk of surgical site infections” (81% to 97% correct pre and post intervention, *P* = .074).

**Table 2.** Module survey responses

Module	Outcome/question	Sample size	Correct/positive responses pre-intervention	Correct/positive responses post-intervention	Statistical test/statistic	*P*-value	Interpretation
Module 1: Clinical impact of PAL presence	Most reported penicillin allergies are true hypersensitivity reactions (T/F)	31	87%	84%	χ^2^ = 0, df = 1	1.00	No significant change
	Penicillin allergies are lifelong (T/F)		87%	94%	χ^2^ = .50, df = 1	.48	No significant change
	PALs increase risk of surgical site infection (T/F)		81%	97%	χ^2^ = 3.20, df = 1	.074	Trend toward significance, not significant
Module 2: Allergy history documentation	Prior education on gathering a comprehensive drug allergy history (Yes/No)	25	24%	NA	–	–	Descriptive only
	Preparedness to gather a comprehensive allergy history (10-point Likert)		Median = 7	Median = 9	WSR V = 0	<.01	Significant improvement
	SmartPhrase user-friendly (Yes/No)		NA	92%	–	–	High acceptability
Module 3: Risk stratification algorithm	Prior education on prescribing antibiotics in patients with drug allergies (Yes/No)	17	47%	NA	–	–	Descriptive only
	Preparedness to assess severity of penicillin allergy (10-point Likert)		Median = 6	Median = 9	WSR V = 8	<.01	Significant improvement
	Preparedness to prescribe surgical antibiotic prophylaxis (10-point Likert)		Median = 5	Median = 8	WSR V = 0	<.01	Significant improvement
	Cefazolin can be considered in patient with penicillin anaphylaxis (T/F)		76%	88%	χ^2^ = .25, df = 1	.62	Not significant; high baseline knowledge
	Algorithm tool is user-friendly (Yes/No)		NA	88%	–	–	High acceptability

T/F represents True and False; NA represents Not Applicable; df represents degrees of freedom; WSR represents Wilcoxon Signed Rank.

### Allergy history documentation

In module 2 (n = 25), 24% reported receiving education on gathering a comprehensive drug allergy prior to this QI initiative. Of those who participated, 92% found the allergy history tool created for this initiative to be user-friendly. For the question: “Do you feel prepared to gather a comprehensive drug allergy history?”, there was a statistically significant improvement in preparedness scores (median change 7 to 9 pre and post intervention, *P* < .01).

For module 3 (n = 17) there were 19 total responses providing unpaired descriptive statistics. Forty-seven percent of participants reported receiving education on antibiotic prescribing in patients with PAL prior to this QI initiative. There was a statistically significant improvement in preparedness scores across the questions assessing preparedness to assess allergy severity (median improved from 6 to 9 pre intervention and post intervention, *P* < .01) and antibiotic prophylaxis selection (median improved from 5 to 8 pre intervention and post intervention, *P* < .01). For the question: “Cefazolin can be considered in a patient with a history of anaphylaxis to penicillin” 76% vs 88% responded correctly, pre versus post module, *P* = .62. Lastly, 88% of participants found the risk-stratification algorithm tool is user-friendly.

### Inductive thematic analysis

The analysis identified three overarching themes; theme 1: opportunities for tool optimization, theme 2: expanding implementation, access, and accessibility, and, theme 3: educational impact and value. Theme 1 highlighted needs for standardized decision-making, improved Epic SmartPhrase usability, better workflow integration, and more structured documentation, along with added dropdowns and clearer labels. Theme 2 focused on scaling across sites, access to pharmacist support, and improved accessibility (eg, closed captioning). Theme 3 reflected enhanced understanding of allergy history-taking and the strong educational value of the modules.

## Discussion

This study found that educational modules improved healthcare providers’ preparedness to obtain comprehensive drug allergy histories, assess penicillin allergy severity, and manage PAL in ambulatory surgical settings requiring SAP, consistent with prior evidence supporting SAP initiatives.^5,6^ Module 1 did not show statistical significance, which may reflect pre-existing knowledge on the clinical implications of PALs.^7^ Module 2 significantly improved preparedness to gather comprehensive drug allergy histories (*P* < .01). Given that confidence is central to behavior change, improved preparedness may support better uptake of allergy history-taking practices.^8,9^ Module 3 significantly improved preparedness to assess penicillin allergy severity and select appropriate SAP (*P* < .01).

Prior evidence supports these findings. A scoping review of ASP educational interventions found that workplace-integrated education can improve behavior change and clinical outcomes.^6^ For example, Koch et al., found that structured online interdisciplinary education and active learning strategies can enhance ASP confidence.^5^ However, through our work we found limited evaluation of user experience; therefore, high acceptability of the Epic SmartPhrase (92%) and the modified ACCEPT algorithm (88%) support their application for multidisciplinary adoption. Thematic analyses identified an improved understanding of allergy history-taking and educational value as key facilitators. Suggested improvements included enhancing the Epic SmartPhrase for workflow integration, expanding to additional sites, adding closed captioning, and increasing perioperative pharmacist support.

This study has several limitations. The quasi-experimental design limits causal inference, though paired analyses improve internal validity. Voluntary participation may introduce self-selection bias, and module 3 had a smaller sample size due to exclusion of staff not involved in antibiotic ordering or verification, reducing statistical power. Predominantly closed-ended surveys limited response depth, partially offset by comment fields. As a single-center ambulatory study, generalizability may also be limited. Although we assessed preparedness and knowledge, future interrupted time-series analysis will evaluate the impact of implementation on prescribing patterns.

Overall, this QI initiative demonstrated that structured tools, algorithms, and targeted education can improve clinician preparedness and confidence in managing PALs for SAP. High acceptability and positive feedback support the potential for sustainable implementation and broader ASP efforts.
